# STORMy Interactions: Gaze and the Modulation of Mimicry in Adults on the Autism Spectrum

**DOI:** 10.3758/s13423-016-1136-0

**Published:** 2016-08-09

**Authors:** Paul A. G. Forbes, Yin Wang, Antonia F. de C. Hamilton

**Affiliations:** 1 Institute of Cognitive Neuroscience, University College London, London, UK; 2 Division of Psychology, New York University, New York, NY USA

**Keywords:** Social cognition, Autism, Mimicry, Gaze

## Abstract

Mimicry involves unconsciously imitating the actions of others and is a powerful and ubiquitous behavior in social interactions. There has been a long debate over whether mimicry is abnormal in people with autism spectrum conditions (ASC) and what the causes of any differences might be. Wang and Hamilton’s (2012) social top-down response modulation (STORM) model proposed that people with ASC can and do mimic but, unlike neurotypical participants, fail to modulate their mimicry according to the social context. This study used an established mimicry paradigm to test this hypothesis. In neurotypical participants, direct gaze specifically enhanced congruent hand actions as previously found; in the ASC sample, direct gaze led to faster reaction times in both congruent and incongruent movements. This result shows that mimicry is intact in ASC, but is not socially modulated by gaze, as predicted by STORM.

People often unconsciously copy each other’s actions, and this mimicry has been described as a powerful social glue (Chartrand & van Baaren, 2009). Individuals with a diagnosis of autism spectrum conditions (ASC) have impairments in social communication and interaction (American Psychiatric Association, 2013) that may include differences in mimicry (Edwards, 2014). A recent theory proposed that these mimicry differences may be due to difficulties in using social cues such as eye contact to modulate mimicry (Wang & Hamilton, 2012). The current paper aimed to test this hypothesis.

The social top-down response modulation (STORM) model (Wang & Hamilton, 2012) proposes that social cues determine whether a particular action should be mimicked. Extensive evidence demonstrates that mimicry is modulated according to the social context. For example, people are more likely to mimic when they feel socially excluded (Lakin, Chartrand, & Arkin, 2008), when interacting with attractive people (van Leeuwen, Veling, van Baaren, & Dijksterhuis, 2009), and those they like (Stel et al., 2010). Although these studies show effects in real-life mimicry over the course of minutes, rapid modulation of mimicry by social cues can be measured using stimulus-response compatibility paradigms (Brass, Bekkering, & Prinz, 2001).

During a typical stimulus-response compatibility experiment, participants observe an action and make either a congruent (e.g., observe hand opening, perform hand opening) or incongruent (e.g., observe hand closing, perform hand opening) prespecified action. Responses to congruent actions are faster than those to incongruent actions, and this congruency effect is taken as a measure of the tendency to mimic. Like real-world mimicry, the congruency effect is modulated by social cues, including eye gaze (Wang, Newport, & Hamilton, 2010), emotional facial expressions (Rauchbauer, Majdandžić, Hummer, Windischberger, & Lamm, 2015), social priming (Leighton, Bird, Orsini, & Heyes, 2010; Wang & Hamilton, 2013) and beliefs about the model’s animacy (Liepelt & Brass, 2010). STORM accounts for these effects by claiming that all mimicry is subject to top-down modulation from a variety of social cues.

When applied to ASC, STORM proposes that mimicry itself is intact but that people with ASC do not use social cues to modulate their mimicry behavior. A number of studies have indirectly supported STORM by showing that neither prosocial priming (Cook & Bird, 2012) nor emotional facial expressions (Grecucci et al., 2013) enhance mimicry in ASC. Moreover, Vivanti and Dissanayake (2014) found that preschoolers without ASC imitated more frequently following direct gaze, whereas age-matched children with ASC imitated to the same extent following direct and averted gaze.

This article aimed to test STORM more directly in a sample of adults with ASC using an established eye-contact mimicry effect. Gaze cues, such as eye contact, provide a foundation for social cognition (Hamilton, 2016). They exert a mixture of arousal, attentional, and social effects on the observer and facilitate downstream information processing, including joint attention, ostensive communication, and mimicry (Böckler, Timmermans, Sebanz, Vogeley, & Schilbach, 2014; Mundy, Kim, McIntyre, Lerro & Jarrold, 2016). An insensitivity to gaze cues has been widely reported in ASC (Senju & Johnson, 2009). However, the exact nature of this insensitivity and its consequences on downstream information processing remain less well characterized.

A series of studies have shown that eye contact modulates mimicry in neurotypical adults (Wang & Hamilton, 2014; Wang et al., 2010) and have outlined the brain mechanisms responsible (Wang, Ramsey, & Hamilton, 2011). In these studies, participants saw either direct or averted gaze before performing a prespecified action that was either congruent (mimicry) or incongruent (not mimicry) with an action on the screen. The results showed three effects—a main effect of congruency (congruent responses are faster than incongruent ones), a main effect of gaze (responses are faster following direct compared to averted gaze), and an interaction between gaze and mimicry (direct gaze enhances congruent, but inhibits incongruent, responses). We consider different hypotheses in reference to each of these effects.

First, previous studies have consistently demonstrated that the basic mechanisms of mimicry are intact in ASC (Bird, Leighton, Press & Heyes, 2007), so we predicted a main effect of congruency. Second, people with ASC seem to process and use eye contact atypically (Senju & Johnson, 2009). If all gaze processing mechanisms are abnormal in ASC, neither congruent nor incongruent responses should be affected by gaze. Finally, STORM predicts that *only* the interaction between gaze and mimicry should be absent in ASC. Participants with ASC can mimic and may be sensitive to gaze as an alerting stimulus, but do not use gaze as a social cue to control mimicry. Our study aimed to test these hypotheses.

## Method

### Participants

Twenty-seven neurotypical adults and twenty-six participants with ASC were recruited from the UCL Institute of Cognitive Neuroscience’s autism@icn participant database. We aimed for a sample size of 25 or more participants. The final sample size was determined by the availability of the participants on autism@icn database during the testing period. Groups were matched on age, gender, handedness, and verbal and performance IQ using either the *Wechsler Adult Intelligence Scale* (WAIS-III UK; Wechsler, 1999a) or *Wechsler Abbreviated Scale of Intelligence* (WASI-II, Wechsler, 1999b; see Table 1). ASC participants had a diagnosis of Asperger’s syndrome (21), autism (3), or autism spectrum disorder (2) from an independent clinician.

**Table 1:** A comparison of the full sample

	ASC (*n* = 26)		NT (*n* = 27)		*t* test
	Mean (*SD*)	Range	Mean (*SD*)	Range	*p* value
Age (years)	28 (7)	18–48	27 (6)	18-40	*p* = .50
Fullscale IQ	120 (12)	95–152	124 (12)	99-151	*p* = .44
Verbal IQ	123 (13)	100–155	125 (12)	98-150	*p* = .85
Performance IQ	114 (13)	87–132	118 (13)	85-148	*p* = .34
ADOS: total	8 (3)	4–17			
ADOS: communication	3 (2)	0-6			
ADOS: social interaction	6 (2)	2–11			
Gender	4 F; 22 M		5 F; 22 M		
Handedness	3 L; 23 R		3 L; 24 R		

ASC participants were assessed on Module 4 of the *Autism Diagnostic Observation Schedule* (ADOS-G, Lord et al., 2000; ADOS-2, Lord et al., 2012) by a trained researcher with research-reliability status. Seven participants met the ADOS classification for autism, eleven for autism spectrum, and eight did not meet the classification for either autism or autism spectrum. Seven out of these eight reached the cut-off for autism spectrum on either the Communication or Reciprocal Social Interaction subscale, and all eight had a clear diagnostic history from an independent clinician. All participants were financially reimbursed and gave written informed consent. All procedures were approved by the UCL Research Ethics Committee.

### Procedure

**Fig. 1:**
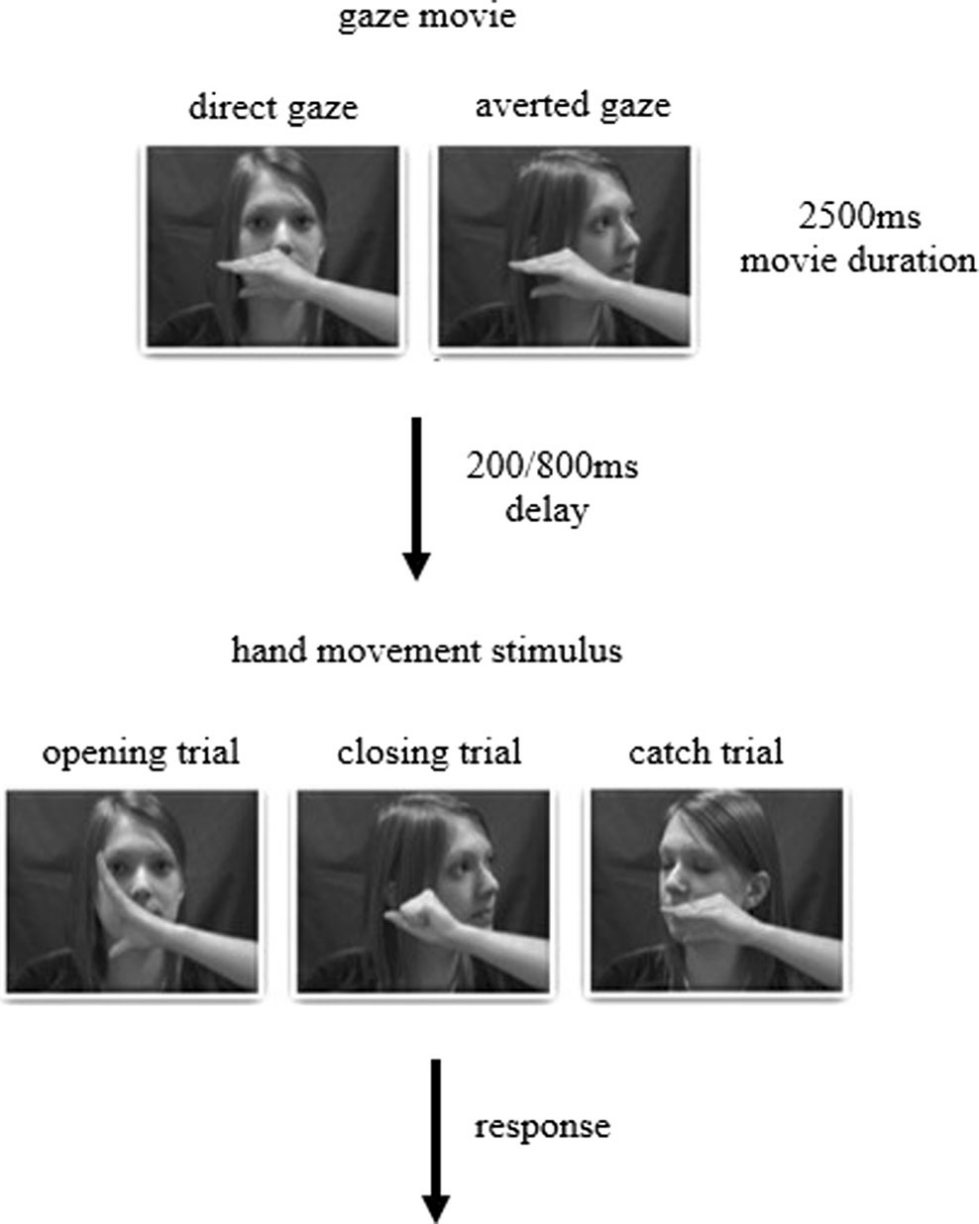
Example of the stimuli used and trial sequence. *Note*. Adapted from Wang et al. (2010)

Participants came into the lab as part of an autism@icn research day in which they completed a number of studies. For this study, they sat approximately 70 cm from the projector screen with their right elbow resting on the desk in front of them and their right hand in a semi-open position. To measure reaction times (RTs), a Polhemus electromagnetic marker was attached to the inside of their right thumb and index finger. Participants completed Wang et al.’s (2010) stimulus-response compatibility paradigm exactly as in their study (see Fig. 1). At the beginning of each block, participants were instructed to either open (opening block) or close (closing block) their hand as quickly as possible when they saw the actor’s hand move, regardless of whether the actor opened or closed her hand on each trial. Thus, for each trial, the participants’ prespecified movements were either the same as the observed action (congruent trials; e.g., observe hand opening, perform hand opening) or the opposite of the observed action (incongruent trials; e.g., observe hand closing, perform hand opening).

Before each trial a fixation cross appeared on the screen for 300 ms. This was followed by a video that started with a female actor facing away from the viewer with her eyes closed and left hand in a static position across her face. She then opened her eyes and either moved her head toward the viewer (direct gaze) or turned her head toward her left or right side (averted gaze). Following the head movement, the actor opened or closed her hand after a delay of either 200 ms or 800 ms (see Fig. 1). Videos appeared 50 cm × 43 cm on a 160 cm × 90 cm projector screen.

Participants completed 160 trials split across four blocks: two hand opening and two hand closing blocks presented alternately and randomized across participants. Within each block, 20% of trials were “catch” trials, during which the actor’s hand remained static. Participants were told to keep their hand static during these trials (i.e., not make the prespecified movement). Participants were given approximately 5 minutes of practice before the experiment, during which they completed a shortened hand opening and hand closing block, and were made familiar with the catch trials.

Data were recorded in MATLAB and video presentation controlled using the Cogent toolbox. Analysis of hand aperture velocity was identical to that used by Wang et al. (2010) and allowed RTs to be calculated.

## Results

### Error rates

Because participants moved either too fast (<50 ms), too slow (>1,000 ms), did not move at all, or made the wrong prespecified movement, 1.72% of trials were excluded. An independent-samples *t* test revealed that there were no significant differences in proportion of trials excluded between neurotypical (NT) and ASC participants (*p* > .250).

### Neurotypical participants

One NT participant’s mean RT was over 3 *SD*s from the mean and so was removed from the final analysis. Removal ensured the normality of the data, but did not disrupt the matching between the groups. RTs were analyzed using a two-way (gaze: directed/averted; congruency: congruent/incongruent) repeated-measures ANOVA, which revealed a significant main effect of congruency, *F*(1, 25) = 47.3, *p* < .001, η_p_^2^ = 0.654, with faster responses when making congruent as opposed to incongruent actions, and a significant interaction between congruency and gaze, *F*(1, 25) = 7.28, *p* = .012, η_p_^2^ = 0.225. Post hoc *t* tests showed that congruent responses were marginally significantly faster when preceded by direct gaze compared to averted gaze, *t*(25) = -1.73, *p* = .097, *d* = -0.117, but this was not the case for incongruent responses, *t*(25) = 1.24, *p* = .227, *d* = 0.081.

### ASC participants

The same analysis was applied to the ASC group’s RTs and revealed a main effect of gaze, *F*(1,25) = 7.05, *p* = .014, η_p_^2^ = 0.220, with faster responses following direct gaze, and congruency, *F*(1, 25) = 72.23, *p* < .001, η_p_^2^ = 0.743, with faster responses when making congruent as opposed to incongruent responses. There was no significant interaction between gaze and congruency, *F*(1, 25) = 0.014, *p* > .250, η_p_^2^ = 0.001.

### Group comparison

To explore group differences, RTs were analyzed using an ANOVA with gaze and congruency as within-subject factors and group as a between-subject factor. This revealed a significant main effect of congruency, *F*(1, 50) = 120.98, *p* < .001, η_p_^2^ = 0.708, and group *F*(^1, 50)^ = 13.26, *p* = .001, η_p_^2^ = 0.210, with neurotypical participants responding faster than ASC participants, and a marginally significant main effect of gaze, *F*(1, 50) = 3.96, *p* = 0.052, η_p_^2^ = 0.073, with a trend to faster responses following direct gaze. The interactions between gaze and group, *F*(1, 50) = 4.03, *p* = 0.050, η_p_^2^ = 0.075, gaze and congruency, *F*(1, 50) = 3.19, *p* = .080, η_p_^2^ = 0.060, and, gaze, congruency and group, *F*(1, 50) = 3.02, *p* = .088, η_p_^2^ = 0.057, were all approaching significance. The interaction between congruency and group, *F*(1, 50) = 2.57, *p* = .115, η_p_^2^ = 0.049, was not significant.

A key measure of the tendency to mimic is the congruency effect calculated as the mean RT to incongruent trials minus mean RT to congruent trials. However, as mean RT increases, the congruency effect also increases (Press, Bird, Flach, & Heyes, 2005). Thus, when testing for modulators of this congruency effect, it is important to control for the confounding influence of mean RT (Butler, Ward, & Ramsey, 2015). To deal with the slower mean RT in the ASC group, we calculated a percentage congruency effect (PCE) relative to overall mean RT for each participant using the following equation:

$$ \mathrm{Percentage}\kern0.28em \mathrm{C}\mathrm{ongruency}\kern0.28em \mathrm{E}\mathrm{ffect}\left(\mathrm{P}\mathrm{C}\mathrm{E}\right)=\frac{\mathrm{Mean}\kern0.28em \mathrm{Incongruent}\kern0.28em \mathrm{R}\mathrm{T}-\mathrm{Mean}\kern0.28em \mathrm{C}\mathrm{ongruent}\kern0.28em \mathrm{R}\mathrm{T}}{\mathrm{Overall}\kern0.28em \mathrm{Mean}\kern0.28em \mathrm{R}\mathrm{T}}\times 100. $$

**Fig. 2:**
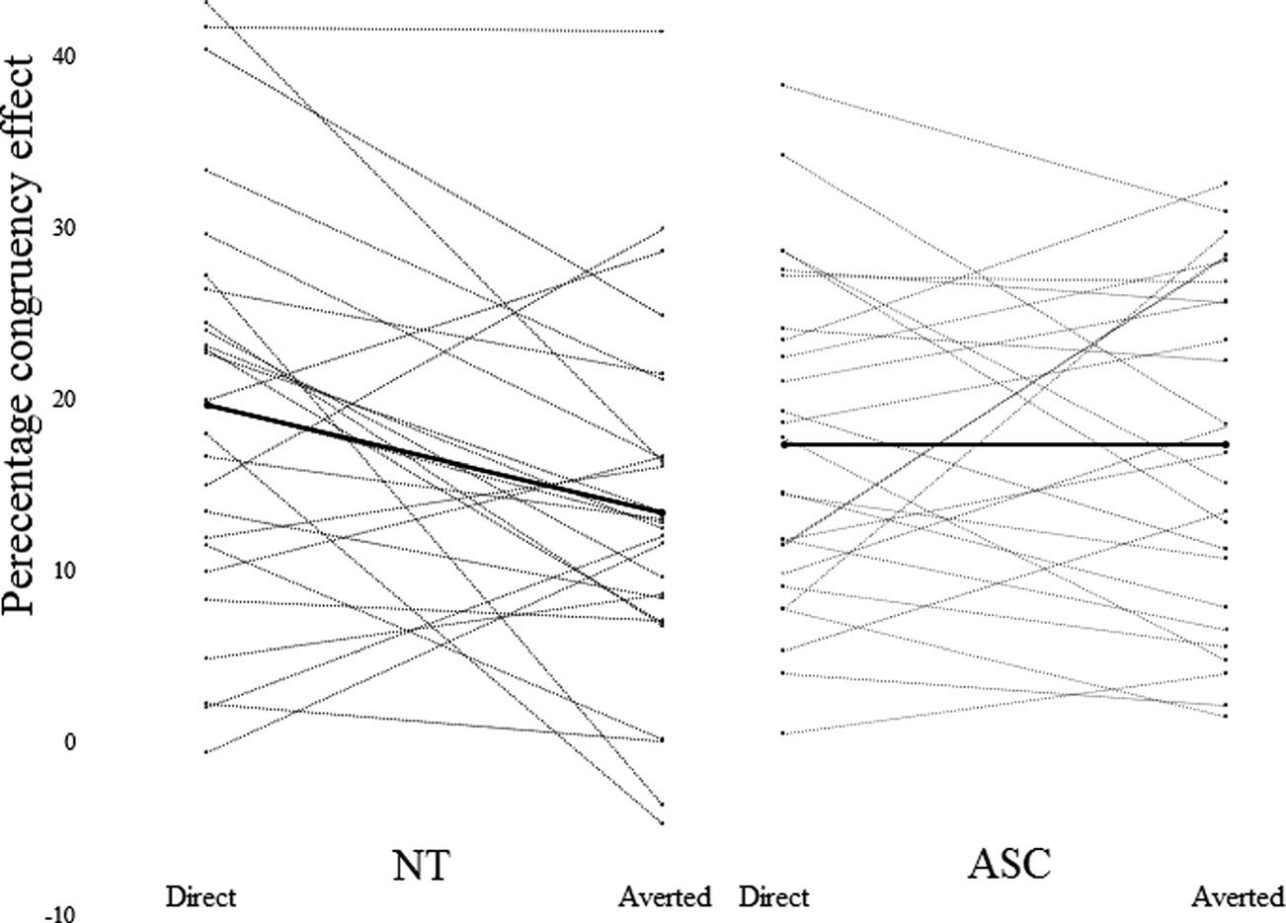
PCE for directed and averted gaze for each NT (*n* = 26) and ASC (*n* = 26) participant; means are in the thicker lines

This expressed how much faster participants’ RTs were for congruent compared to incongruent trials in each gaze condition, relative to their overall mean RT. Using the PCE as a measure of the tendency to mimic, we can compare the influence of gaze on mimicry between groups (see Fig. 2).

PCEs were analyzed using an ANOVA with gaze as a within-subjects factor and group as a between-subject factor. This revealed no main effect of group, *F*(1, 50) = 0.153, *p* = .698, η_p_^2^ = 0.003, but a significant main effect of gaze, *F*(1, 50) = 4.25, *p* = .045, η_p_^2^ = 0.078, and, importantly, there was a significant interaction between gaze and group, *F*(1, 50) = 4.28, *p* = .044, η_p_^2^ = 0.079. Post hoc *t* tests revealed the PCE was greater in the direct gaze condition in NT participants, *t*(25) = 2.70, *p* = .012, *d* = 0.559, but there was no difference in PCE between the two gaze conditions in the ASC group, *t*(25) = -0.007, *p* > .250, *d* = 0.001.

### Subgroup analysis

Because eight ASC participants did not meet the cut-off for an ADOS classification of either autism spectrum or autism, the same analysis was conducted for the eighteen participants who did meet cut-off. These eighteen did not differ from the NT group on age, verbal, or performance IQ (see Table 2).

**Table 2:** A comparison of the subgroups

	ASC (*n* = 18)		NT (*n* = 26)		*t* test
	Mean (*SD*)	Range	Mean (*SD*)	Range	*p* value
Age (years)	28 (5)	20–37	26 (6)	18–40	*p* = .62
Fullscale IQ	119 (14)	95–152	124 (13)	99–151	*p* = .35
Verbal IQ	123 (14)	100–155	125 (12)	98–150	*p* = .65
Performance IQ	112 (14)	87–132	118 (14)	85–148	*p* = .28
ADOS: total	10 (3)	7–17			
ADOS: communication	3 (1)	2–6			
ADOS: social interaction	7 (2)	4–11			
Gender	2 F; 16 M		5 F; 21 M		
Handedness	2 L; 16 R		3 L; 23 R		

**Fig. 3:**
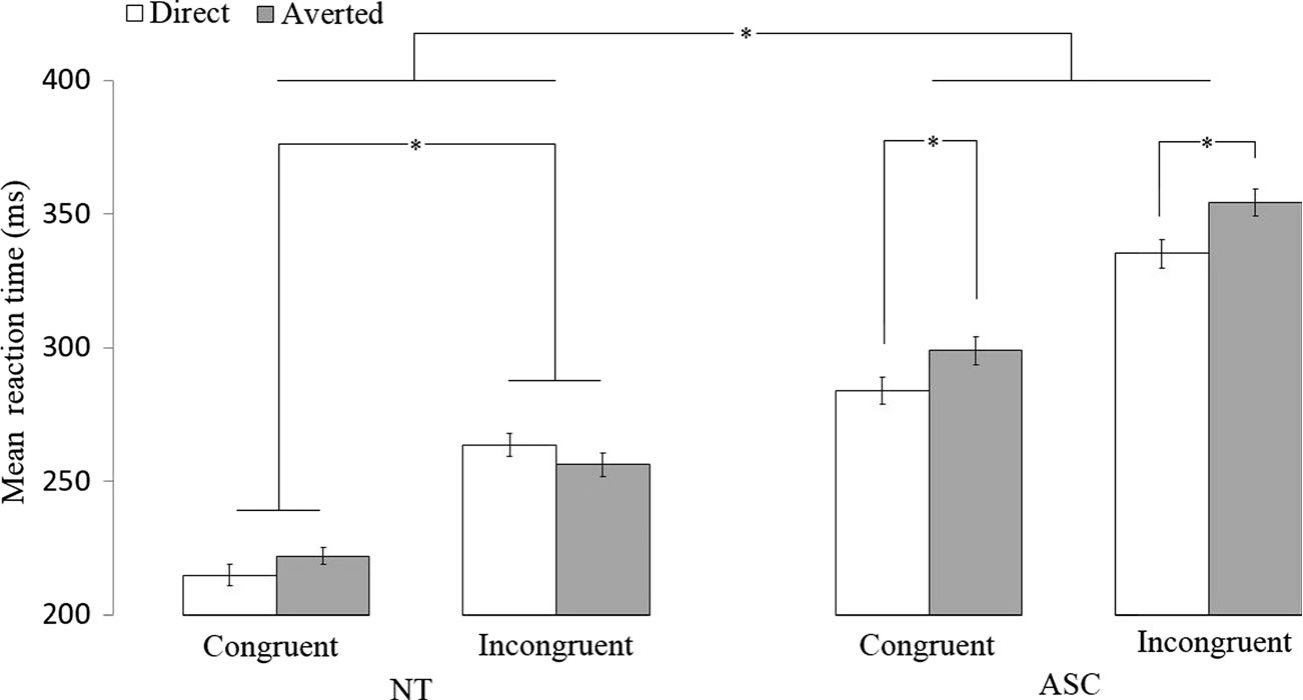
Mean RT (+/- *SEM*) for congruent and incongruent trials for direct and averted gaze for NT participants (*n* = 26) and those reaching cut-off for an autism spectrum or autism classification on ADOS (*n* = 18)

Analysis of RTs revealed a main effect of gaze, *F*(1, 42) = 6.31, *p* = .016,, η_p_^2^ = 0.131, congruency, *F*(1, 42) = 93.04, *p* < .001, η_p_^2^ = 0.689, and group, *F*(1, 42) = 13.21, *p* = .001, η_p_^2^ = 0.239. The interaction between gaze and group, *F*(1, 42) = 6.40, *p* = .015, η_p_^2^ = 0.132, and the interaction between gaze, group, and congruency, *F*(1, 42) = 4.24, *p* = .046, η_p_^2^ = 0.092, was significant (see Fig. 3). The interactions between group and congruency, *F*(1, 42) = 1.45, *p* = .235, η_p_^2^ = 0.033, and, gaze and congruency, *F*(1, 42) = 1.30, *p* > .250, η_p_^2^ = 0.030, were not significant. Analysis of PCE revealed no main effect of gaze, *F*(1, 42) = 2.06, *p* = .159, η_p_^2^ = 0.047, or group, *F*(1, 42) < .001, *p* > .250, η_p_^2^ < 0.001, but, as with the whole group analysis, showed a significant interaction between gaze and group, *F*(1, 42) = 4.84, *p* = .033, η_p_^2^ = 0.103. Post hoc *t* tests revealed that the PCE was greater in the direct gaze condition in NT participants, *t*(25) = 2.70, *p* = .012, *d* = 0.559, but there was no difference in PCE between the direct and averted gaze conditions in ASC, *t*(25) = -0.542, *p* > .250, *d* = -0.132 (see Fig. 3).

## Discussion

Neurotypical and ASC adults performed a stimulus-response compatibility task measuring the tendency to mimic in the presence or absence of direct gaze. Neurotypical adults showed a stronger mimicry effect following direct gaze. Adults with ASC mimicked but did not show this specifically social enhancement of mimicry by gaze. Instead, they showed faster RTs for both congruent and incongruent responses following direct gaze. We discuss our findings in terms of STORM and current theories of gaze processing in ASC.

### STORMy interactions

ASC participants demonstrated a reliable congruency effect that suggests the basic mechanisms responsible for imitative responses are intact in ASC (Edwards, 2014). Our data did not support the hypothesis that all gaze processing is disrupted in ASC (Senju & Johnson, 2009), as a consistent main effect of gaze was found. All responses were faster after direct, compared to averted, gaze in ASC. In neurotypical participants, direct gaze enhanced congruent responses (i.e., mimicry), but slowed incongruent responses, resulting in no overall effect of gaze. The general gaze effect suggests people with ASC may use gaze as an alerting or attentional signal and are not entirely immune to signals from the eyes.

STORM predicted that if social top-down response modulation is abnormal in ASC, then the interaction between gaze and congruency found in neurotypical participants would be absent in ASC. The results support STORM because, in ASC, the tendency to mimic was not enhanced following direct, compared to averted, gaze. This lack of social modulation of mimicry by gaze is consistent with previous studies showing that neither prosocial priming (Cook & Bird, 2012) nor emotional facial expressions (Grecucci et al., 2013) enhance mimicry in ASC. The findings also corroborate evidence from preschoolers with ASC who imitated to the same extent in direct and averted gaze conditions, unlike their neurotypical peers who imitated more following direct gaze (Vivanti & Dissanayake, 2014).

### Gaze processing in ASC

Our findings are consistent with previous studies, which suggest that gaze cues do not have a social effect on downstream information processing in ASC (Böckler et al., 2014; Mundy et al., 2016). Participants with ASC differentiated between direct and averted gaze as all responses were faster in the direct gaze condition. This suggests that direct gaze may have had an attentional or alerting effect in participants with ASC. However, direct gaze did not have a specifically social effect on mimicry, as was found in neurotypical participants. This generic, nonsocial effect of gaze cues was also found by Ristic et al. (2005), who showed that individuals with ASC were sensitive to gaze direction when it was spatially informative (i.e., it predicted the location of a cue). However, unlike neurotypical individuals, those with ASC were no longer sensitive to gaze direction when it was spatially uninformative (i.e., gaze direction predicted the location of the cue at chance levels). So, there may be sensitivity to gaze in ASC as a generic spatial cue (or as an attentional or alerting stimulus, as we found in this study), but not sensitivity to gaze cues as specifically social stimuli.

Recent neuroimaging studies further support the hypothesis that gaze cues do not have the same social impact in ASC. In neurotypical participants, direct compared to averted gaze resulted in increased activity in areas involved in theory-of-mind processing, such as medial prefrontal cortex, temporoparietal junction, and, posterior superior temporal sulcus (von dem Hagen, Stoyanova, Rowe, Baron-Cohen & Calder, 2013). Furthermore, activation of medial and orbital prefrontal regions has been shown to be positively correlated with gaze duration in neurotypicals (Kuzmanovic et al., 2009); in ASC, however, these classic “social brain” areas were preferentially activated by *averted* as opposed to direct gaze (Georgescu et al., 2013; von dem Hagen et al., 2013). Together, these behavioral and neuroimaging data suggest individuals with ASC may show some sensitivity to gaze cues but might not reap all the social effects of these cues.

## Conclusion

People with ASC can unconsciously copy the actions of others, but do not use important social cues, such as gaze, to determine when and what to mimic. Participants with ASC were sensitive to direct gaze as an attentional or alerting stimulus, but did not use gaze as a specifically social stimulus to modulate their mimicry.
